# Feasibility, safety, and economic consequences of using minimal flow anaesthesia by Maquet FLOW-i equipped with automated gas control

**DOI:** 10.1038/s41598-021-99648-4

**Published:** 2021-10-08

**Authors:** Yusuf Z. Colak, Hüseyin I. Toprak

**Affiliations:** Department of Anaesthesiology and Reanimation, Medical Faculty, İnönü University, Malatya, 44280 Turkey

**Keywords:** Environmental impact, Clinical trials

## Abstract

Low fresh gas flow rates are recommended because of their benefits, however, its use is limited due to associated risks. The main purpose of this study was to investigate whether 300 mL of fresh gas flow that practised with automated gas control mode is applicable and safe. The second aim is to show that automated mode can provide economic benefits. Sixty hepatectomy cases who suitable criterias were included to cohort study in three groups as prospective, sequential, observational. An operating room were allocated only for this study. 300 mL fresh gas flow with automated mode (groupA3), 600 mL fresh gas flow with automated mode (groupA6) and, 600 mL fresh gas flow with manually (groupM6) was applied. Patients’ respiratory, hemodynamic parameters (safety), number of setting changes, O_2_ concentration in the flowmeter that maintained FiO_2_:0.4 during the low flow anaesthesia (feasibility) and comsumption data of anaesthetic agent and CO_2_ absorber (economical) were collected and compared. p < 0.05 was accepted as statistical significance level. No significant differences were detected between the groups in terms of demographic data and duration of operation. Safety datas (hemodynamic, respiratory, and tissue perfusion parameters) were within normal limits in all patients. O_2_ concentration in the flowmeter that maintained FiO_2_:0.4 was statistically higher in groupA3 (92%) than other groups (*p* < 0.001) but it was still within applicable limits (below the 100%). Number of setting changes was statistically higher in groupM6 than other groups (*p* < 0.001). The anaesthetic agent consumption was statistically less in groupA3 (*p* = 0.018). We performed fresh gas flow of 300 mL by automated mode without deviating from the safety limits and reduced the consumption of anaesthetic agent. We were able to maintain FiO_2_:0.4 in hepatectomies without much setting changes, and we think that the automated mode is better in terms of ease of practise.

## Introduction

A significant portion of inhalation agents, which constitute the main components of anaesthetic drug costs (20–25%), are released into the atmosphere via the waste gas system without being metabolized by the patient. The amount of waste gas is directly proportional to the amount of fresh gas flow (FGF)^1^. All volatile agents have a greenhouse gas effect on the atmosphere, which is highest for desflurane. The total annual global emissions of volatile anaesthetics in 2014 were calculated to be equivalent to the CO_2_ emissions of one-third of passenger cars in Switzerland, with approximately 80% stemming from desflurane^2^. For this reason, low flow anaesthesia (LFA) and FGF < 1 L/min have been recommended by anaesthesiologists in recent years to minimize wastage^3^. During inhalation anaesthesia, LFA improves the dynamics of inhaled anaesthetic gas, increases mucociliary clearance, maintains body temperature, and reduces water loss. Furthermore, according to the 2019 Worldwide Medical Trends Report, inflation in health products increases three times more than in other products^4^. The use of LFA provides savings of up to 75%^5^. Thus, LFA is not only beneficial for patients but also economical and environmentally friendly. Similar comments can be made for minimal flow anaesthesia (MFA: 0.25–0.5 L/min). However, the routine use of FGF below 1 L/min is avoided for a variety of reasons, including differences in the concentrations inhaled by the patient and agent concentration in FGF adjusted by the vapourizer, the need for more frequent vapourizer and rotameter adjustments, vapourizer dial setting increment variability, and the risks associated with LFA (accidental hypoxia, hypercapnia, inadequate depth of anaesthesia, and the accumulation of potentially toxic trace gases). By using modern anaesthesia machines with advanced monitoring of respiratory gas concentrations, LFA can be used in almost all patients without increased risk^6,7^, and automated low flow anaesthesia (ALFA) is easy to use. ALFA has brought LFA within the realm of everyday practice. The use of ALFA obviates the need for frequent vapourizer and FGF adjustments that may distract busy clinicians, especially during the induction period. First, the anaesthesiologist selects a target alveolar concentration (FAt) of inhaled anaesthetic and a target O_2_%. Next, proprietary software algorithms guide agent and carrier gas administration to attain the targets with the lowest waste^8^.

O_2_ consumption can be calculated with a simplified Brody formula (VO = 10 × [BW]^¾^). However, the simpler estimate of oxygen consumption that has been used is 3–5 mL/kg/min for adults^9^. According to these calculations, the O_2_ consumption per minute of a 70 kg adult is approximately 250 mL Therefore, we presumed that 300 mL FGF will be sufficient to meet the O_2_ requirements of adults and that the lowest FGF that can be practised in the Maquet Flow-i device is 300 mL via the automated gas control (AGC) mode. In our previous study, we showed that 600 mL FGF with AGC can be used without deviating from the safety limit of FiO_2_:0.4 in donor hepatectomies, and it reduced the costs by 38% compared with 1200 mL FGF with AGC^10^.

The main purpose of this study was to investigate whether 300 mL of FGF in the AGC mode is applicable and safe in the normoxic range. The second aim was to show that the AGC mode with 300 mL of FGF can provide more economic and ecological benefits than the AGC or manual mode with 600 mL of FGF.

## Materials and methods

Similar to our previous study (10), we selected adult patients scheduled for hepatectomy procedures under general anaesthesia who had an American Society of Anesthesiologists (ASA) physical status score of 1–2 with generally similar body weights (BW) to obtain a standardized patient population. The sample size, 20 patients in each group, was calculated to detect a difference in desflurane consumption of 30% with a standard deviation (SD) of ± 4 mL using an alpha level of 0.05 and a power of 0.80.

After ethical approval was received from the institutional review board (Malatya Clinical Research Ethics Committee) for our prospective, sequential, observational cohort study (NCT03465475), sixty hepatectomy patients with informed consent, aged 18–65 years who met the criteria were included in the study at Inonu University between 2020 and 2021 in accordance with the relevant guidelines and regulations. Patients with diabetes mellitus, those who had cardiovascular and pulmonary diseases, those who had a body mass index < 20 or > 30, those who did not want to participate in the study, and those with urgent cases were excluded from the study. Patients undergoing laparoscopic procedures were not included in the study because these procedures could impair oxygenation. An operating room and an anaesthesia machine (Maquet Flow-i 40, Solna, Sweden® anaesthesia machine with a circular breathing system and a 700 mL CO_2_ absorber canister) were allocated only for this study population, and we chose a sequential design, so we were able to calculate the total consumption of each group precisely. The anaesthesia and surgery teams that worked in the room during the study did not change. No premedications were ordered. The ages, heights, BWs, and sexes of the subjects were recorded. During the operation, electrocardiography (ECG), heart rate (HR/min), systolic arterial pressure (SAP, mmHg), diastolic arterial pressure (DAP, mmHg), mean arterial pressure (MAP, mmHg), peripheral oxygen saturation (SpO_2_, %), body temperature (°C) (Carescape B650, GE Healthcare, Helsinki, Finland®), Pleth variable index (PVI) (to evaluate intravascular volume status), non-invasive peripheral haemoglobin (SpHb-g/dL), perfusion index (PI), oxygen reserve index (ORI), and patient state index (PSI) (to evaluate level of anaesthesia) (Root; Masimo, Irvine, CA, USA®) were monitored for patient safety. In addition, a regional cerebral oximetry (rSO_2_L-rSO_2_R) measurement was made from the left and right frontal regions with the cerebral near-infrared spectroscopy (NIRS) method (INVOS 5100C; Medtronic, MN, USA®). The values were recorded at baseline; after anaesthesia induction; post-intubation; post-intubation plus 10 min; and post-intubation plus 1, 2, 3, 4, 5, and 6 h. Blood gas samples for PaO_2_ were taken at the beginning of LFA (PO_2_1) and just before LFA was terminated (PO_2_2).

After preoxygenation, anaesthesia was induced with thiopental 5–8 mg/kg, fentanyl 1–2 mcg/kg, lidocaine 1 mg/kg, and vecuronium 0.1 mg/kg, and when adequate anaesthesia depth was reached, an endotracheal tube of appropriate size was inserted. Invasive arterial monitoring was carried out in all patients. When we considered the oxygen requirement (approximately 3 mL/kg/min), leaks in the breathing circuit, and safety range, it was thought that 300 mL FGF would be sufficiently safe. Hedenstierna stated in his review that an inspired oxygen concentration of 30%–40%, or even less, should suffice if the lungs are kept open^11^. Therefore, for the first 20 patients, 300 mL FGF in the AGC mode (group A3) was applied; for the second 20 patients, 600 mL FGF in the AGC mode (group A6) was applied; and for the third 20 patients, 600 mL FGF (group M6) was applied manually. The target values in all patients were specified as FiO_2_:0.4, 1 minimal alveolar concentration (MAC) on the desflurane vapourizer, and the respective FGF stated above. The AGC tool also allows the user to select 1 out of 9 different speeds with which the target FAdesf can be reached (with 9 being the fastest speed). The AGC mode at 6 speeds was applied to achieve the FAdesf target in groups A3 and A6. In group M6, these targets were reached manually, and an FGF rate of 6 L/min (3–3:oxygen–air) and a dial setting of 1 MAC on the desflurane vapourizer were performed. On achieving the target MAC value on the respiratory gas monitor, the flow rate was reduced to 600 mL/min. The vapourizer was adjusted to maintain 1 MAC during surgery at intervals of two minutes, and the flowmeter was adjusted for FiO_2_:0.4 during the same period. Every change that needed to be made to settings was counted (NOSC) (to evaluate feasibility). For all patients, intraoperative mechanical ventilation was initiated with a tidal volume of 8 mL/kg, with the rate was titrated to maintain end-tidal carbon dioxide (etCO_2_) of 35–40 mmHg. Analgesia management was achieved with remifentanil infusion. When the amount of inspired CO_2_ ≥ 3 mmHg, the CO_2_ absorbent was changed. The times to reach the targets were recorded in all groups to evaluate feasibility. The required O_2_ concentration in the flowmeter to obtain the FIO_2_:0.4 ratio (cFlowO_2_) during LFA/MFA was recorded every ten minutes in all patients to determine whether we were staying within the limits of safety and feasibility. At the end of the operations, the total operation durations and amounts of anaesthetic gas consumed were recorded individually for each patient (by the anaesthesia device), and after the surgeries for each group were completed, the empty desflurane and CO_2_ absorbent bottles were calculated for economic consequences.

### Statistical analysis

Quantitative data used in the study were summarized as median (min–max). The conformity of the quantitative variables to the normal distribution was examined using the Shapiro–Wilk test. In terms of quantitative variables, the Kruskal Wallis-H test was used to determine whether there was a statistical difference between independent groups. After the Kruskal Wallis-H test, whether there was a statistically significant difference between the groups was examined with the Conover test. p < 0.05 was accepted as statistical significance level. In the analysis, web-based softwares (“KruskalWallis” and "IAY: Istatistiksel Analiz Yazilimi”) were used developed by İnönü University Faculty of Medicine Biostatistics and Medical Informatics Dept^12,13^.

## Results

Throughout the study, the data of 60 patients (20 patients from each group) were collected. No significant differences were detected among the groups in terms of age, BW and operation duration (Table 1). No significant differences were detected among the groups in terms of gender (p = 0.736).

**Table 1:** The age, BW, and operation duration between the groups (median, min–max).

	Group A3 (n = 20)	Group A6 (n = 20)	Group M6 (n = 20)	p
Age (year)	27 (18–47)	28 (22–51)	28.5 (19–43)	0.813
BW (kg)	66 (44–80)	61 (42–79)	67 (48–81)	0.416
Op. duration (min)	343 (257–540)	356 (247–521)	345 (250–580)	0.959

No significant differences were detected among the groups in terms of haemodynamic, respiratory or tissue perfusion parameters (SpO_2_, NIRS) (p > 0.05). The haemodynamic, respiratory, and tissue perfusion parameters were within normal limits in all patients at all times. SpO_2_, rSO_2_L and rSO_2_R values did not fall below the basal values during the surgeries. GroupM6 post-intubation plus 10 min ORI value was statistically higher than groupA3 and A6 (p = 0.017). SpO_2_, rSO_2_L, and rSO_2_R, which are indicators of tissue oxygenation, and the ORI values are given in Table 2.

**Table 2:** Indicators of tissue oxygenation, SpO_2_, rSO_2_L, rSO_2_R, and ORI (median. min–max).

	GroupA3 (n = 20)	GroupA6 (n = 20)	GroupM6 (n = 20)	p
**Basal**				
SpO_2_ (%)	99 (97–100)	99 (97–100)	98 (97–99)	0.295
rSO_2_L (%)	71 (60–85)	71.5 (55–83)	69.5 (61–84)	0.992
rSO_2_R (%)	72 (54–84)	71.5 (55–83)	70 (58–82)	0.741
ORI	0 (0–0.06)	0 (0–0.15)	0 (0–0.05)	0.793
**AAI**				
SpO_2_ (%)	100 (99–100)	100 (99–100)	100 (99–100)	0.429
rSO_2_L (%)	75 (62–93)	74.5 (55–95)	73.5 (64–91)	0.850
rSO_2_R (%)	79 (55–90)	75 (60–87)	74.5 (62–89)	0.490
ORI	0.5 (0.3–0.85)	0.51 (0.19–1)	0.545 (0.24–1)	0.926
**Pin**				
SpO_2_ (%)	100 (98–100)	100 (98–100)	100 (98–100)	0.253
rSO_2_L (%)	82 (69–95)	82.5 (61–95)	78.5 (69–93)	0.750
rSO_2_R (%)	86 (65–95)	83.5 (57–92)	80 (67–92)	0.349
ORI	0.41 (0.21–0.57)	0.44 (0.14–0.78)	0.41 (0.19–1)	0.609
**PIn10 min**				
SpO_2_ (%)	98 (97–100)	99 (97–100)	99 (97–100)	0.197
rSO_2_L (%)	79 (59–93)	76 (59–93)	76 (64–85)	0.850
rSO_2_R (%)	78 (53–92)	75.5 (59–84)	72.5 (63–90)	0.477
ORI	0.24^b^ (0–0.52)*	0.21^b^ (0.04–0.69)*	0.33 (0.1–0.52)	***0.017***
**PIn-1 h**				
SpO_2_ (%)	98 (96–100)	99 (96–100)	98.5 (97–100)	0.215
rSO_2_L (%)	82 (65–95)	79.5 (66–95)	83.5 (67–95)	0..417
rSO_2_R (%)	84 (56–95)	79 (61–90)	81.5 (65–93)	0.438
ORI	0.29 (0–0.69)	0.32 (0–0.62)	0.325 (0.04–0.58)	0.851
**PIn-2 h**				
SpO_2_ (%)	99 (97–100)	99 (97–100)	99 (96–100)	0.490
rSO_2_L (%)	81 (64–95)	81.5 (66–95)	81 (70–94)	0.928
rSO_2_R (%)	82 (63–95)	81 (55–89)	82 (64–94)	0.602
ORI	0.46 (0–1)	0.42 (0.23–1)	0.44 (0.28–1)	0.852
**PIn-3 h**				
SpO_2_ (%)	99 (97–100)	99 (97–100)	98.5 (98–100)	0.256
rSO_2_L (%)	80 (61–95)	81.5 (70–95)	81 (65–95)	0.911
rSO_2_R (%)	81 (59–95)	81 (61–91)	80 (64–93)	0.852
ORI	0.62 (0.11–1)	0.42 (0.25–0.96)	0.47 (0.31–1)	0.512
**PIn-4 h**				
SpO_2_ (%)	100 (98–100)	100 (97–100)	99 (97–100)	0.188
rSO_2_L (%)	83 (61–95)	80.5 (65–95)	80 (64–96)	0.527
rSO_2_R (%)	84 (58–94)	81 (56–90)	80 (64–92)	0.262
ORI	0.81 (0.18–1)	0.59 (0.3–1)	0.54 (0.39–1)	0.847
**PIn-5 h**				
SpO_2_ (%)	100 (97–100)	100 (97–100)	99.5 (98–100)	0.752
rSO_2_L (%)	83.5 (68–95)	79 (66–95)	80.5 (67–94)	0.138
rSO_2_R (%)	84 (62–95)	82 (18–92)	78 (65–91)	0.166
ORI	0.69 (0.31–1)	0.74 (0.34–1)	0.7 (0.41–1)	0.895

AAI, after anaesthesia induction; Pin, post intubation.

*a: different according to the A6 group, b: different according to the M6 group. Statistically significant “p” values ​​are in bold and italic.

No significant differences were detected among the groups in terms of PaO_2_. Except for one patient in group A3, all PaO_2_ values were over 100 mmHg. This patient’s PaO_2_ value was 97.9 mmHg (Table 3).

**Table 3:** PaO_2_ Values (mmHg, median, min–max).

	GroupA3	GroupA6	GroupM6	p
PaO_2_1	177 (97.9–210)	173.5 (110–199)	176 (121–192)	0.945
PaO_2_2	172 (139–192)	181.5 (156–199)	174.5 (145–194)	0.143

None of the patients had bleeding that impaired the haemodynamics and required blood transfusion. The body temperatures were in the normal range (min–max: 36.1–37.2 °C). The PSI levels were between 25 and 50 in all patients, and no differences were detected among the groups (p = 0.810).

cFlowO_2_ that maintained FiO_2_:0.4 and provided adequate oxygenation during LFA/MFA was 92% (min 81%–max 100%) in group A3, 63% (min 57%–max 67%) in group A6, and 66% (min 59%–max 70%) in group M6, and there was a statistically significant difference between group A3 and the other groups (p < 0.001). Two patients in group A3 needed 100% oxygen from the flowmeter for a short time during the operation to maintain FiO_2_:0.4. A very short-lived (less than 1 min) very high FGF (> 10 L/min) ensured the target FiO_2_ in groups A3 and A6. The target FiO_2_ was attained within 1–2 min, and 1 MAC end-tidal gas concentration was reached in approximately 5–6 min in all groups (p = 0.632). As a result, the target set in all groups was sustained, but the vapourizer and flowmeter were adjusted multiple times (more than 10 changes) to reach target values in groupM6. There was a significant difference in terms of NOSC values between group M6 and the other groups (p < 0.001).

There was a difference of a few millilitres between the empty anaesthetic agent (AA) bottles counted and the data from the anaesthesia machine, so we used the data from the anaesthesia machine. The median AA consumption was 57.1 mL in groupA3 and statistically less then the other groups (p = 0.018) (Table 4). Total AA consumption was 1305 mL in group A3, 1680 mL in group A6, and 1540 mL in group M6. The amount of CO_2_ absorber used was 26 kg in group A6, 24.5 kg in group M6 and 36.2 kg in group A3. There was a significant difference in terms of CO_2_ absorber values between group A3 and the other groups (p < 0.001).

**Table 4:** AA consumption in groups (mL, median, min–max).

	Group A3	Group A6	Group M6	*p*
AA Cons	57.1^a,b^ (37.4–113.8)*	75.4 (49.1–139.6)	76.8 (25.4–116.8)	***0.018***

*a: different according to the A6 group, b: different according to the M6 group. Statistically significant “p” values ​​are in bold and italic.

## Discussion

Oxygenation and the depth of anaesthesia are two main concerns in LFA and MFA. The O_2_ level in the atmosphere is 21%, and the PaO_2_ in the blood of humans is approximately 100 mmHg. Considering oxygen as a drug, many studies have shown that both low and high oxygen levels are risky^10^. Therefore, oxygenation should be performed as close to the normal levels as possible. We believe that oxygen demand should be determined and applied according to the patients’ demands under anaesthesia. The most commonly used formula for this purpose is Brody’s formula^14^. The average weight of the subjects in all groups was similar, approximately 65 kg. Basal metabolic O_2_ consumption was calculated as approximately 250 mL/min. In group A3, FGF was set at 300 mL; in group A6 and group M6, FGF was set at 600 mL. When the O_2_ and air in FGF were adjusted to maintain FiO_2_:0.4, these FGFs delivered more oxygen than required (250 mL O_2_) for all patients. As mentioned in our findings, cFlowO_2_ values to achieve the FIO_2_:0.4 ratio showed that there was a safety range even at 300 mL FGF (group A3 cFlowO_2_ = 92%) and it was still within applicable limits (below the 100%). The haemodynamic and oxygenation parameters (SpO_2_ and rSO_2_) included in our study did not exceed the safety limits in any patient, and the SpO_2_, rSO_2_, and ORI values did not fall below the basal values (measured in room air) during the operation. Group M6 post-intubation plus 10 min ORI value was statistically higher than the other groups but this difference is clinically insignificant. These findings support our calculations as given above. In addition, PSI values ​​showed an adequate depth of anaesthesia in all groups. Therefore, we can say that we had adequate oxygen and AA delivery in all groups, and we were able to maintain this situation safely throughout the operation.

Two patients needed 100% O_2_ for a short time, and one patient’s PO_2_1 was 97.9 mmHg in group A3, but the SpO_2_ and NIRS values of these patients were 97%, 98%, and 97% and L79%-R79%, L80%-R80%, and L71%-R76%, respectively, during the same time period. However, the BW of these patients was close to the group mean. Considering that we eliminated body weight differences, we speculate that this situation may depend on breathing system leaks. System leaks become very important at this low flow rate. The Maquet Flow-i40 anaesthesia machine has advanced sensors and warning systems and does not allow the use of the AGC mode if the leakage amount is above 150 mL/min. If possible, there should be no breathing system leakage when working at such low flows.

We used the same flow rates in group A6 and group M6 and reached the targets via the AGC mode in group A6. The same targets were reached manually in group M6. In the comparison between these two groups, we found no statistically significant difference in haemodynamic, respiratory or consumption data. However, multiple adjustments were needed to achieve the targets in group M6. Additionally, when the absorbent canister was replaced, adjustments were made again to meet the targets, and canister changes were frequent in LFA/MFA. Therefore, the NOSC values ​​were very high in group M6. We have seen that the AGC mode is successful in achieving the targets set at the incident inception and maintaining these targets throughout the operation. Therefore, we believe that the AGC mode is better than manual adjustments in terms of the ease of use.

We set an FGF of 300 mL/min in group A3. According to the modified baker and simionescu classification, this value is approximately equal to MFA. When we performed a similar comparison for group A6 and group M6, the FGF (600 mL/min) was approximately equal to LFA, according to the same classification. LFA saves up to 75% compared to higher FGFs^5^. In our previous study^10^, we compared medium-flow anaesthesia with LFA and achieved a 42% profit in AA cost, in the present study, profit was ~ 33%. The profit decreased as the compared values decreased, but still a significant decrease was found in the A3 group in terms of AA cost. In the present study, this comparison is between 600 mL FGF and 300 mL FGF but would be much more saving at an FGF higher than 600 mL. As expected, CO_2_ absorbent consumption was higher in group A3, but total cost (AA + CO_2_ absorbent) of group A3 was less than the other groups since CO_2_ absorbent was very inexpensive compared to the AA.

The impact of inhaled AA on the overall global environmental pollution has been known for more than a decade^15^. Lowe and Ernst, while describing the closed system anaesthesia practice they applied in 1981, stated that the reduction in fluorocarbon emissions from operating rooms is an additional ecological benefit^16^. Desflurane may have a greater potential environmental impact than the other drugs because of the higher concentrations required and its intrinsic properties as a greenhouse gas (GHG) (9). The GHG impact of desflurane is 15 times that of isoflurane and 20 times that of sevoflurane on a MAC-hour basis when applied in an O_2_/air mixture. (3). FGF is the main determinant of the amount of waste gas released into the atmosphere. Therefore, it should focus on the amount of FGF to reduce the environmental impact of waste AA^17^. In the last few years, we have started to feel the effects of global warming seriously. If the use of LFA is not limited to clinical studies and becomes widespread all over the world, it is obvious that there will be environmental gains as well as economic gains. ALFA is easy to use and may increase LFA/MFA use by reducing concerns about oxygenation and the depth of anaesthesia. In addition, less pollution of the operating room air is another benefit in terms of personal safety. Gauger et al. stated that spontaneous abortion rates increased in anaesthetists who were exposed to waste gases more^18^.

Our study had some limitations. First, minimal circuit leaks were inevitable. Second, the anaesthesia machine did not measure the amount of CO_2_ absorbent consumed, so we had to choose a sequential design, but no significant differences were detected among the groups in terms of demographic data and operating durations.

## Conclusions

With our high-tech anaesthesia devices and monitors, using LFA/MFA in almost all patients is a logical option to improve our environment and relationship with nature, ensure our future, and minimize costs. Using ALFA may reduce some concerns about hypoxia and anaesthesia depth during operation, increase low flow rate usage and provide an ease of use. In the present study, we performed FGF of 300 mL in AGC mode without deviating from the safety limits and reduced the AA cost. We were able to maintain FiO_2_:0.4 in patients undergoing hepatectomies without many setting changes, and we think that the AGC mode is better than manual adjustments in terms of ease of use.
